# Multiple pulmonary Aspergillus fumigatus cysts and cavities that disappeared with anti‐fungal agents

**DOI:** 10.1002/rcr2.327

**Published:** 2018-08-01

**Authors:** Miwako Saitou, Tomoko Suzuki, Katsunao Niitsuma

**Affiliations:** ^1^ Department of Infectious Disease and Pulmonary Medicine Aizu Medical Center, Fukushima Medical University Aizuwakamatsu Japan

**Keywords:** Aspergillus fumigatus, cavities, check‐valve mechanism, cysts, disappeared

## Abstract

A 26‐year‐old man with a 10‐year history of asthma was admitted to our hospital with a six‐week history of dry cough and slight fever. We observed the left pneumothorax with multiple cysts and cavities and performed partial cystectomy of the left upper lung. Y‐shaped mycelia were detected in the resected tissue, and Aspergillus fumigatus was cultured. Pulmonary aspergillosis was diagnosed from the histopathological and bacteriological findings, and the patient demonstrated positive immunological reactions to A. fumigatus. After administration of an intravenous antifungal agent for one month and an oral antifungal agent for another three months, all cysts and cavities disappeared. Aspergillus infection usually results from saprophytic growth within pre‐existing cavities. In this case, multiple cysts and cavities may have been caused by *Aspergillus*, possibly through a check‐valve mechanism.

## Introduction

Pulmonary involvement with *Aspergillus* spp. is varied and depends on the underlying pulmonary and immune status. Mycetomas develop from secondary colonization of pre‐existing lung cavities. Mycosis may develop multiple cavitary lesions in immunodeficient lungs 1. Invasive aspergillosis and chronic progressive pulmonary aspergillosis (CPPA) mostly affect patients with altered immune status 2.

We encountered a case where *Aspergillus fumigatus* caused multiple cysts, cavitary lesions, and an allergic state. After antifungal treatment, all cysts and cavities disappeared. This represents a very unusual form of pulmonary involvement with *A. fumigatus.*

## Case Report

A 26‐year‐old man was admitted to our hospital in the month of September a few years ago with a six‐week history of dry cough and two weeks of slight fever and anterior chest pain. The patient also described malaise and a 5‐kg weight loss during the summer. He had no history of smoking and was employed in agriculture, where he had sometimes worked in a humid warehouse**.** The patient had experienced episodes of wheezing dyspnoea since childhood and had been treated with fluticasone inhalation therapy since he was 16 years old. He had seasonal allergic conjunctivitis and many kinds of food allergy (including almond, pumpkin seeds, natto, peanuts, and coffee). However, he did not have any allergic history to any mould, including aspergillus. His serum IgE level was 283 IU/mL.

Physical examination revealed emaciation, with a body mass index of only14.5 kg/m^2^. Body temperature was 37.2°C. Vital signs were normal. Chest examinations and cardiac examination revealed no abnormalities. Neither cyanosis nor clubbing of the fingers was evident.

**Figure 1:**
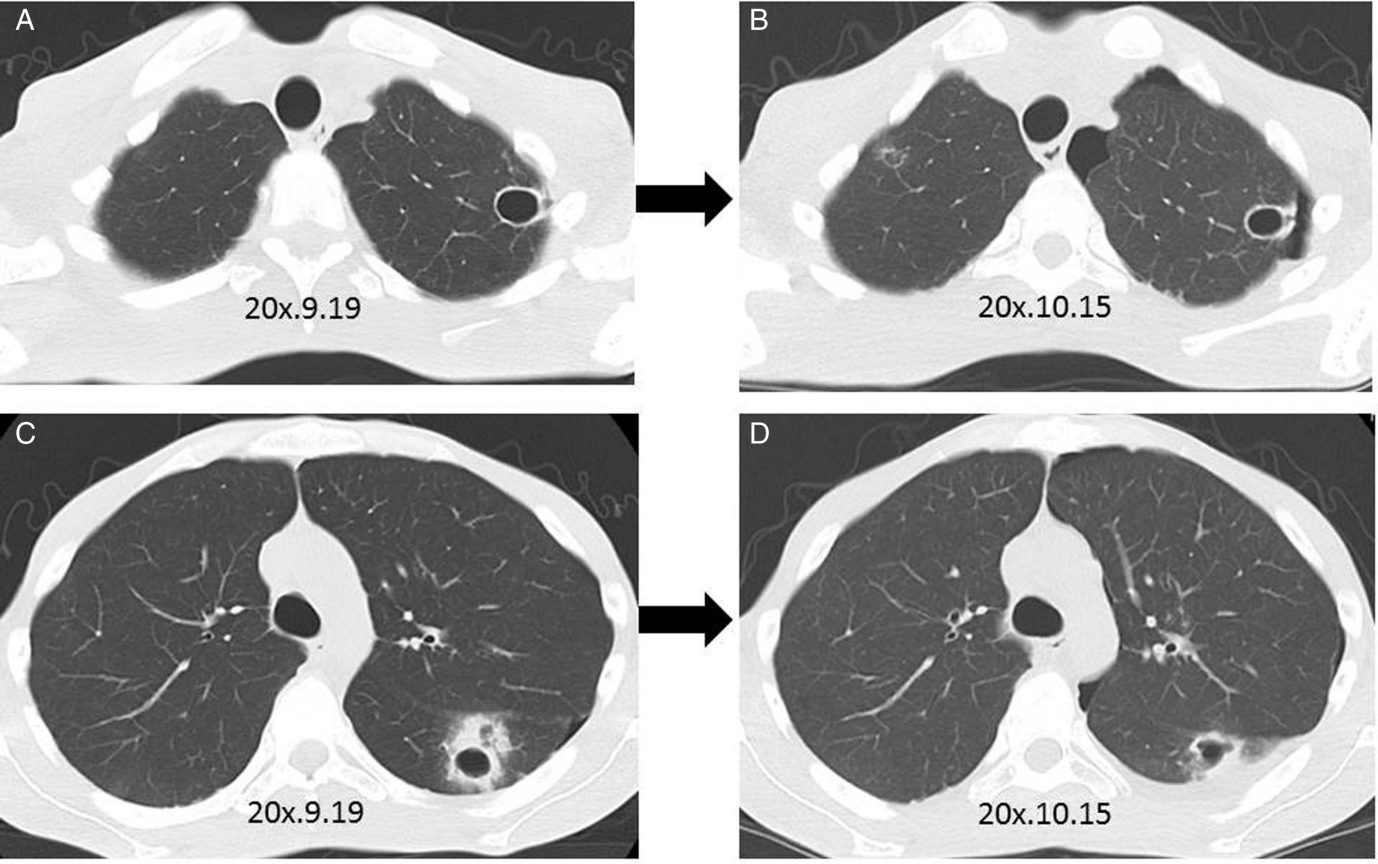
(A) Chest computed tomography shows a cavity in left upper field. (B) The cavity in left upper field is growing, adheres to the visceral pleura, and ruptures. A cyst in right upper field appears. (C) A cavity in left lower field is seen. (D) The cavity changes shape and adheres to the visceral pleura.

Chest radiography demonstrated cavities in the superior segment of the left upper lobe with infiltration. Chest computed tomography (Fig. 1) showed cavities and a cyst in both lung fields. One of those cavities adhered to the visceral pleura of the superior lung field and had ruptured and caused pneumothorax of the left lung (Fig. 1). As the pneumothorax was exacerbating, he underwent partial resection of the left upper lobe. Bacteriologically, *A. fumigatus* was cultured from resected lung tissue. Pathological findings showed Y‐shaped mycelia in the resected specimen. The wall of the cyst displayed chronic inflammation with granulation tissues and necrosis. Based on the bacteriological and pathological findings, pulmonary aspergillosis was diagnosed.

**Figure 2:**
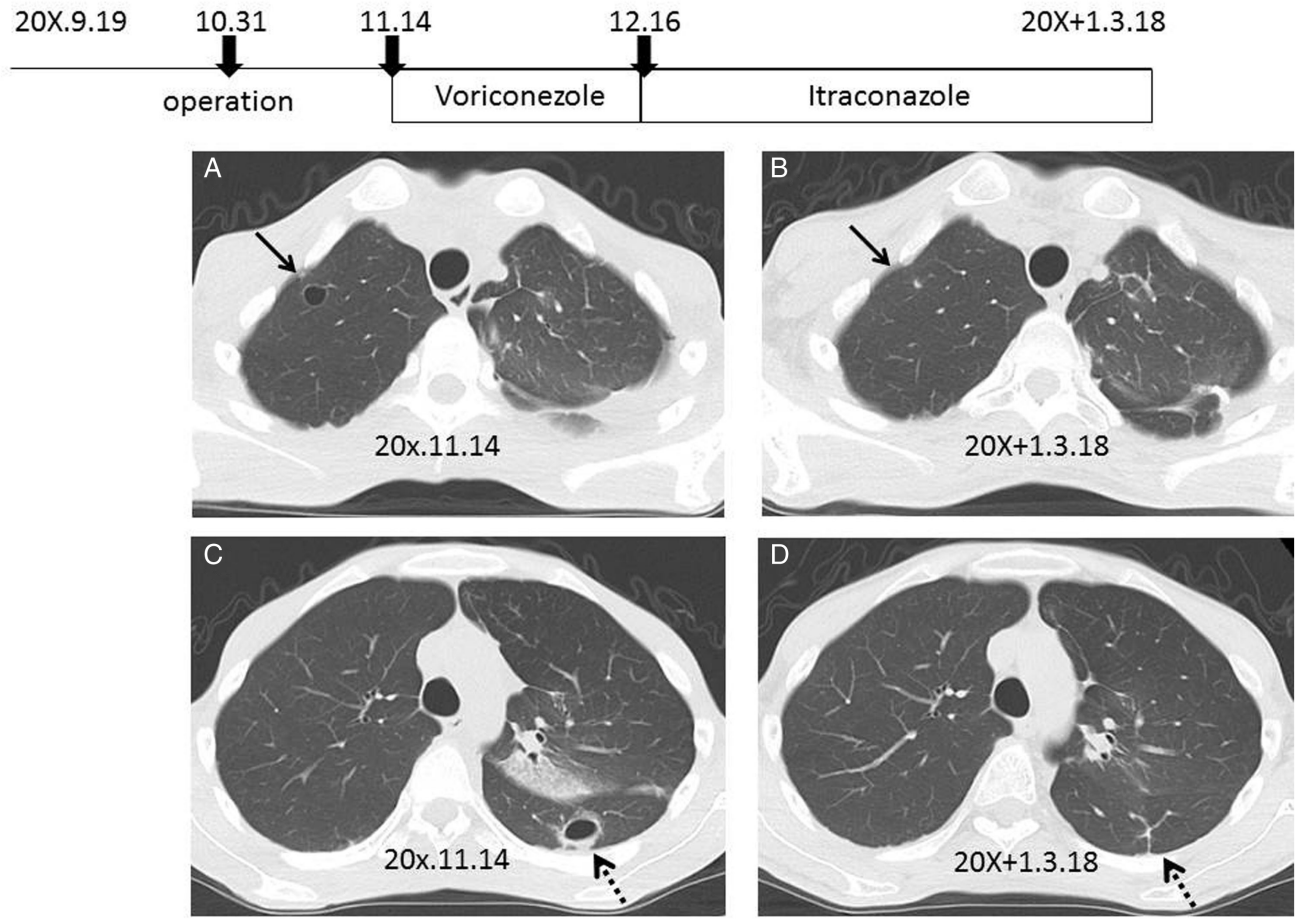
Upper panel shows the clinical course of treatment. (A) Chest computed tomography shows a cyst in the right upper field after partial resection of the left upper lesion. (B) A cyst in right upper field disappeared after antifungal treatment. (C) A cavity in left lower field is seen. (D) The cavity in left lower field became scars after antifungal treatment.

The intravenous antifungal agent voriconazole was administered at 320 mg/day for one month, and he developed pancytopenia and visual impairment. Then, he was treated with oral itraconazole at 400 mg/day for the following two months; after antifungal treatment, his cavities and cyst all disappeared (Fig. 2).

## Discussion

Pulmonary aspergillosis represents a common, potentially lethal opportunistic infection that has four unique forms: simple pulmonary aspergilloma (SPA), CPPA, invasive pulmonary aspergillosis (IPA), and allergic bronchopulmonary aspergillosis 2.

Simple pulmonary aspergilloma and CPPA are considered to result from secondary colonization and saprophytic growth of *Aspergillus* within pre‐existing cavities 2.

Some cases have been described in which pulmonary aspergillosis may have been caused as a side effect of inhaled corticosteroid therapy 3, 4.

In our case, the patient was suffering from bronchial asthma; receiving low‐dose inhalation corticosteroid therapy; and working in a hot, humid cabin environment, and these factors, along with body weight loss during summer, might have contributed to the development of pulmonary aspergillosis.

Generally, the pathogenesis of cavity and cyst formation is not fully clear, but with regard to lung cancer, three mechanisms may lead to the formation of cavities: expectoration of necrotic tumour tissue, invasion of pre‐existing cysts or bullae, and check‐valve mechanisms induced by narrowed airways 5.

In our case, pathological findings identified *Aspergillus* infection granulations in the bronchiolar region, and antifungal therapy led to the disappearance of all cavities and cysts. This outcome suggested that a check‐valve mechanism was involved in the formation of the cavities and cysts in this case.

Only one case in Medline has reported that *Aspergillus* infection might cause cystic lesions through check‐valve mechanisms. No case reports have described the administration of antifungal agents leading to the disappearance of cavities and cysts. This is quite an interesting case.

### Disclosure Statement

Appropriate written informed consent was obtained for publication of this case report and accompanying images.
